# Incidence of treated brain metastases among patients with stage I-III breast cancer: A population-based study

**DOI:** 10.1016/j.breast.2026.104723

**Published:** 2026-02-13

**Authors:** Katarzyna J. Jerzak, Italo Fernandes, Hany Soliman, Bo Zhang, Refik Saskin, Geoffrey Liu, Sunit Das, Arjun Sahgal, Kelvin KW. Chan, Monika Kryzanowska, Rania Chehade

**Affiliations:** a Division of Medical Oncology, Sunnybrook Odette Cancer Centre, University of Toronto, Toronto, Ontario, Canada; b Institute of Medical Sciences, Faculty of Medicine, University of Toronto, Toronto, Ontario, Canada; c Department of Radiation Oncology, Sunnybrook Odette Cancer Centre, University of Toronto, Toronto, Ontario, Canada; d ICES, Toronto, Ontario, Canada; e Division of Medical Oncology and Hematology, Princess Margaret Cancer Centre, University Health Network, Toronto, Canada; f Division of Neurosurgery, St Michael's Hospital, University of Toronto, Toronto, Canada; g Institute of Health Policy, Management and Evaluation, University of Toronto, Toronto, Ontario, Canada

## Abstract

**Background:**

The incidence and risk factors for brain metastases among patients with stage I-III breast cancer remain poorly defined.

**Patients and methods:**

We conducted a population-based cohort study using Ontario health administrative databases to identify patients diagnosed with stage I–III breast cancer between 2009 and 2021. Treatment of brain metastases with surgery or radiation was extracted from the same databases. Patients were stratified by breast cancer subtype: human epidermal growth factor receptor 2 positive/hormone receptor positive (HER2+/HR+), HER2+/hormone receptor negative (HER2+/HR-), HR+/HER2-, and triple-negative breast cancer (TNBC). Primary outcomes were the cumulative incidence of treated brain metastases and time to brain metastasis (TTBM) as defined by the time from primary breast cancer diagnosis to brain metastases treatment.

**Results:**

Among 92,973 patients, 7.9% had HER2+/HR+, 3.5% HER2+/HR−, 54.1% HR+/HER2−, 7.3% TNBC, and 27.2% unknown subtype. Median (IQR) follow-up was 84.2 (50.8–125.2) months. The 12-year cumulative incidence of treated brain metastases was 2.8% in the overall cohort. Among patients with stage III disease, 12-year incidence was 11.8% (HER2+/HR+), 14.3% (HER2+/HR−), 5.9% (HR+/HER2−), and 13.4% (TNBC); corresponding 5-year incidences were 7.5%, 11.2%, and 13.1% for stage III HER2+/HR+, HER2+/HR−, and TNBC, respectively. Among patients with stage III HER2+/HR− and TNBC, median TTBM was 23.3 and 18.0 months, respectively.

**Conclusion:**

Up to 13% of patients with stage III HER2+ or TNBC received treatment for brain metastases within 5 years of diagnosis with early-stage breast cancer. These findings support prospective studies of risk-stratified screening for asymptomatic brain metastases in patients with early-stage breast cancer.

## Highlights

• Incidence of treated brain metastases (BrM) was assessed in a cohort of 92,973 patients with stage I-III breast cancer (BC).
• The incidence of treated BrM was 2.8% in the overall cohort at 12 years.
• In those with stage III triple negative (TN) or HER2+/hormone receptor (HR)- BC this risk was 13.4% and 14.3% at 12 years.
• The shortest median time-to-treatment for BrM was 18.0 months in stage III TNBC and 23.3 months in stage III HER2+/HR- BC.
• These findings support prospective, risk-stratified BrM screening studies for patients with high-risk HER2+ and TN early BC.

## 1 Introduction

Brain metastases are common among patients with metastatic breast cancer [1] and they are associated with significant morbidity and mortality. In a population-based cohort from Ontario, Canada, of 3916 patients with de-novo metastatic breast cancer, 14.0% required radiotherapy for brain metastases with a median (IQR) follow-up of 25.1 months (12.4–40.6) [2]. The highest risk was observed among patients with human epidermal growth factor receptor 2 positive/hormone receptor-negative (HER2+/HR-), HER2+/HR+, and triple-negative breast cancer (TNBC) with a cumulative brain metastases incidence of 34.7%, 28.1%, and 21.9%, respectively [2].

However, the risk of brain metastases among patients who are initially diagnosed with early-stage breast cancer has not been well established. In a literature review of 33 studies, the incidence of brain metastases as the first site of metastatic recurrence per year of median follow-up ranged from .1 to 3.2% [3]. Unlike lung cancer and melanoma, where baseline brain imaging is recommended as part of staging investigations [4,5], guidelines do not recommend brain imaging in asymptomatic patients with early-stage breast cancer. Furthermore, population-based databases frequently lack specific codes for brain metastases, limiting studies in this area.

Symptomatic brain metastases are associated with decreased quality of life, and shorter survival than asymptomatic brain metastasis [6,7]. As screening has not routinely been recommended [8], only about 25% of patients with brain metastases are diagnosed when asymptomatic [ [7], [9], [10]]. Historically, the main treatment of patients with brain metastases consisted of local therapy, such as whole brain radiotherapy (WBRT), stereotactic radiosurgery (SRS), and/or surgery in selected cases [11]. However, new drugs including small molecule tyrosine kinase inhibitors and novel antibody drug conjugates have recently emerged and improved the efficacy of brain metastases treatments [12,13]. Therefore, with improvements in the intracranial efficacy of systemic therapies there is potential for improved outcomes with early detection and intervention for brain metastases.

We used Ontario-wide health administrative data to identify the incidence of treated brain metastases and time to development of brain metastases in patients diagnosed with early-stage breast cancer by subtype and disease stage. Study results will help clarify brain metastases prevalence and risk factors.

## 2 Methods

We conducted a retrospective population-based cohort study using Ontario health administrative data available at the Institute of Clinical Evaluative Sciences (ICES). ICES is an independent, non-profit research institute funded by an annual grant from the Ontario Ministry of Health and Long-Term Care. This study was approved by the research ethics board at Sunnybrook Research Institute, and the requirement for informed consent was waived because ICES is a prescribed entity under Ontario's Personal Health Information Protection Act. We followed the Strengthening the Reporting of Observational Studies in Epidemiology (STROBE) reporting guidelines.

### 2.1 Population

Patients diagnosed with stage I-III breast cancer between January 1st, 2009 and December 31st, 2021 were identified. We then assessed the incidence of brain surgery for malignancies and/or first radiotherapy among these patients. Follow-up data was collected up to December 31st, 2023. Rates of radiation therapy (WBRT with intervention code ‘1AN27JA’ and SRS with intervention code ‘1AN27JX’) were calculated using radiation exposure data captured in the Discharge Abstract Database (DAD), or National Ambulatory Care Reporting System (NACRS) between breast cancer diagnosis and study end. Selected neurosurgery fee code procedures (‘N103’ ‘N115’ ‘N151’) in the Ontario Health Insurance Plan (OHIP) database were used to identify patients who received brain surgery after breast cancer diagnosis. Additionally, patients with a prior cancer diagnosis before the index date or a subsequent diagnosis of a non-breast cancer (including any primary brain tumor, solid tumor, or hematological malignancy) during the study period were excluded. Patients were also excluded if they were non-Ontario residents, were ineligible for OHIP, or if they were under 18 years of age or had died on or before the index date. In this way, we selected patients with a prior diagnosis of breast cancer, who underwent neurosurgical procedures for oncologic indications and/or brain radiotherapy, and had no other cancer diagnoses.

### 2.2 Other variables

We reported on clinical variables including breast cancer subtype (HER2+/HR+, HER2+/HR-, HR+/HER2-, TNBC, and unknown subtype), disease stage (I-III), patient age, residence setting (urban vs rural) [14], VW Elixhauser weighted comorbidity index [15], income status, and index year at diagnosis. American Joint Committee on Cancer (AJCC) disease stage at diagnosis was reported according to the Collaborative Staging Methodology [16].

### 2.3 Outcomes

Primary outcomes were the cumulative incidence of treated brain metastases and time to brain metastasis (TTBM) as defined by the time from primary breast cancer diagnosis to brain metastases treatment. Overall survival (OS), defined as time from treated brain metastases to death due to any cause, was a secondary outcome.

### 2.4 Statistical analysis

The cumulative incidence of treated brain metastases and TTBM were analyzed by breast cancer subtype and stage. Patients with longer follow-up would be expected to have a higher observed incidence of brain metastases; this bias was addressed by measuring cumulative incidences. Patients whose HR and/or HER2 status were not available or incomplete were categorized as having an unknown breast cancer subtype. To identify factors associated with the development of brain metastases, characteristics of patients with and without treated brain metastases were compared. Finally, median survival time from brain metastases to death was calculated. Data were censored for patients who remained alive at the end of the last follow-up date. Categorical variables are presented as a percentage of nonmissing cases and continuous variables are reported as mean (SD) or median (IQR).

Cumulative incidence of treated brain metastases was calculated using the Cumulative Incidence Function (CIF), accounting for the risk of death using a competing risk analysis and described as the incidence of treated brain metastases. Corresponding percentage incidence at 1-, 3-, 5-, 9- and 12-year time points were indicated in tabular format. Characteristics of patients with and without treated brain metastases were compared using a Fine-Gray model. Kaplan-Meier analyses were performed for the time to event endpoints and compared using the log-rank test. A 2-sided *P* < 0.05 was considered statistically significant, with *P* values for difference in medians estimated using a Kruskal-Wallis test and *P* values for categorical values derived from a χ^2^ test. Data were analyzed using SAS Enterprise Guide 8.3 (SAS Institute Inc. Cary, NC, USA).

## 3 Results

From 137,217 patients initially identified, 92,973 were included in the analysis after exclusion criteria were applied (Supplement Fig. 1). The median [interquartile range (IQR)] follow-up time was 84.2 (50.8-125.2) months. Patients with treated brain metastases were younger at breast cancer diagnosis: 53 years (44–62) compared to 61 years (51–71) (p < 0.001). The distribution of breast cancer subtypes was as follows: 7.9% HER2+/HR+, 3.5% HER2+/HR-, 54.1% HR+/HER2-, 7.3% TNBC; and the remaining 27.2% patients had an unknown breast cancer subtype. In terms of staging: 53.5% had stage I, 33.7% had stage II, and 12.8% had stage III disease at initial diagnosis (Table 1).

**Table 1:** Baseline characteristics of patients with stage I-III breast cancer by development of brain metastasis (BrM).

Variable	Label	BrM^a^	No BrM^b^	Total	P Value
Patient No.		N = 2037	N = 90,936	N = 92,973	
Age at BC Diagnosis	Mean (SD)	53.5 (12.8)	61.3 (13.5)	61.1 (13.5)	<.0001
	Median (IQR)	53.0 (44.0-62.0)	61.0 (51.0-71.0)	61.0 (51.0-71.0)	<.0001
	1. 18-40 - n (%)	318 (15.6%)	5121 (5.6%)	5439 (5.9%)	<.0001
	2. 41-60 - n (%)	1137 (55.8%)	38,663 (42.5%)	39,800 (42.8%)	
	3. 61-80 - n (%)	542 (26.6%)	39,177 (43.1%)	39,719 (42.7%)	
	4. >80 - n (%)	40 (2.0%)	7975 (8.8%)	8015 (8.6%)	
Income Quintile and Rurality	1: lowest - n (%)	284 (13.9%)	14,264 (15.7%)	14,548 (15.6%)	.0355
	2 - n (%)	327 (16.1%)	15,698 (17.3%)	16,025 (17.2%)	
	3 - n (%)	394 (19.3%)	15,718 (17.3%)	16,112 (17.3%)	
	4 - n (%)	383 (18.8%)	16,651 (18.3%)	17,034 (18.3%)	
	5: highest - n (%)	419 (20.6%)	18,091 (19.9%)	18,510 (19.9%)	
	6: rural - n (%)	224 (11.0%)	10,366 (11.4%)	10,590 (11.4%)	
	7: missing - n (%)	6 (.3%)	148 (.2%)	154 (.2%)	
Elixhauser Score	1. score<0 - n (%)	424 (20.8%)	17,239 (19.0%)	17,663 (19.0%)	<.0001
	2. score = 0 - n (%)	860 (42.2%)	38,337 (42.2%)	39,197 (42.2%)	
	3. score:1-4 - n (%)	565 (27.7%)	23,325 (25.6%)	23,890 (25.7%)	
	4. score ≥ 5 - n (%)	188 (9.2%)	12,035 (13.2%)	12,223 (13.1%)	
Index Year at BC Diagnosis	2009 - n (%)	209 (10.3%)	5830 (6.4%)	6039 (6.5%)	<.0001
	2010 - n (%)	211 (10.4%)	6449 (7.1%)	6660 (7.2%)	
	2011 - n (%)	191 (9.4%)	6546 (7.2%)	6737 (7.2%)	
	2012 - n (%)	194 (9.5%)	6486 (7.1%)	6680 (7.2%)	
	2013 - n (%)	223 (10.9%)	6723 (7.4%)	6946 (7.5%)	
	2014 - n (%)	187 (9.2%)	7090 (7.8%)	7277 (7.8%)	
	2015 - n (%)	188 (9.2%)	7284 (8.0%)	7472 (8.0%)	
	2016 - n (%)	167 (8.2%)	7548 (8.3%)	7715 (8.3%)	
	2017 - n (%)	144 (7.1%)	7502 (8.2%)	7646 (8.2%)	
	2018 - n (%)	111 (5.4%)	7671 (8.4%)	7782 (8.4%)	
	2019 - n (%)	84 (4.1%)	7889 (8.7%)	7973 (8.6%)	
	2020 - n (%)	81 (4.0%)	6718 (7.4%)	6799 (7.3%)	
	2021 - n (%)	47 (2.3%)	7200 (7.9%)	7247 (7.8%)	
Breast Cancer Subtype	1. TNBC - n (%)	386 (18.9%)	6365 (7.0%)	6751 (7.3%)	<.0001
	2. HER2+/HR+, - n (%)	228 (11.2%)	7149 (7.9%)	7377 (7.9%)	
	3. HER2+/HR-, - n (%)	174 (8.5%)	3108 (3.4%)	3282 (3.5%)	
	4. HR+/HER2-, - n (%)	550 (27.0%)	49,736 (54.7%)	50,286 (54.1%)	
	5. Unknown - n (%)	699 (34.3%)	24,578 (27.0%)	25,277 (27.2%)	
Breast Cancer Stage	I - n (%)	307 (15.1%)	49,411 (54.3%)	49,718 (53.5%)	<.0001
	II - n (%)	835 (41.0%)	30,508 (33.5%)	31,343 (33.7%)	
	III - n (%)	895 (43.9%)	11,017 (12.1%)	11,912 (12.8%)	

Note: % is column percentage; p value for the comparison among the 2 treatment groups.

Legend: BC = breast cancer; HR = hormone receptor; TNBC = triple negative breast cancer.

a BrM treated with brain surgery and/or radiation (whole brain radiotherapy and/or stereotactic radiosurgery).

b No BrM (no local brain-directed therapy).

### 3.1 The incidence of treated brain metastasis

**Fig. 1:**
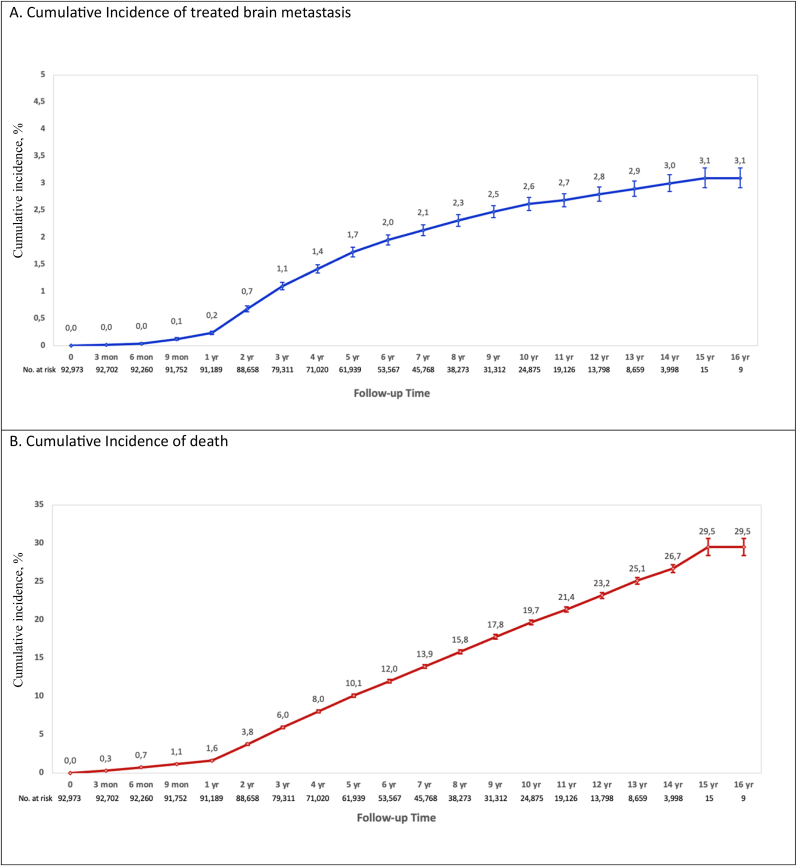
Cumulative incidence of treated brain metastasis and competing risk of death in the overall cohort of patients diagnosed with stage I-III breast cancer.

**Fig. 2:**
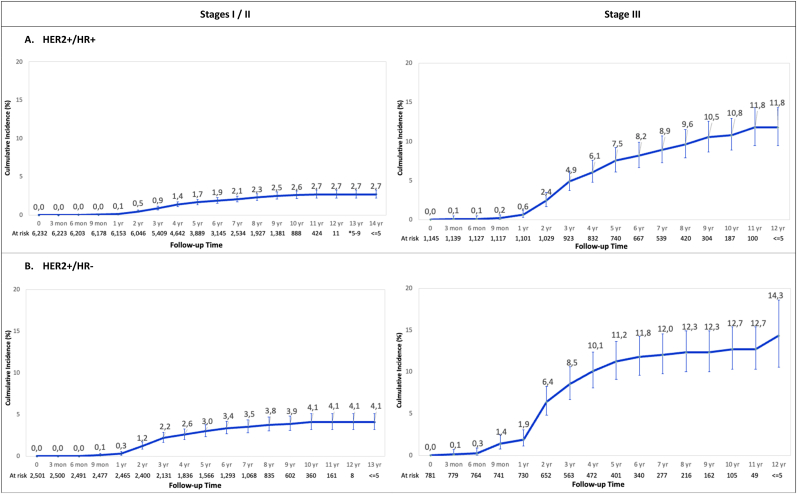

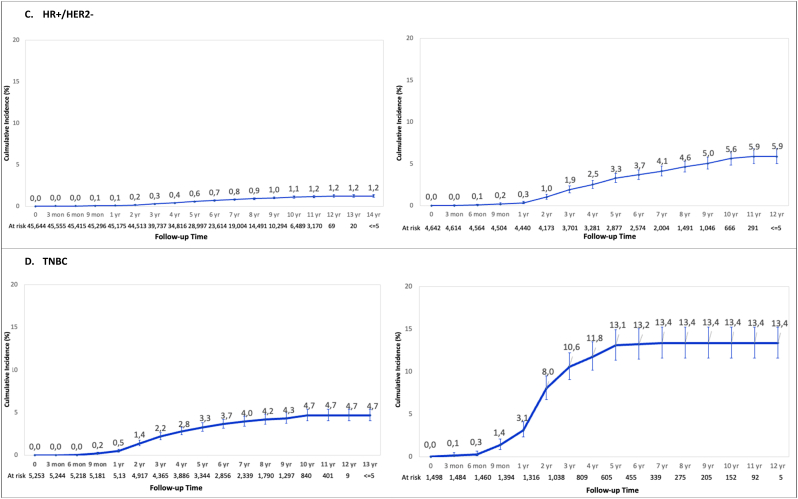
Cumulative incidence of treated brain metastasis by breast cancer subtype and stage at diagnosis Legend: HR = hormone receptor; TNBC = triple negative breast cancer.

In total, 2.2% (n = 2037) of patients underwent treatment for brain metastases (Table 2). The cumulative incidence of treated brain metastases in the overall cohort at 12 years was 2.8%. The cumulative incidence of death at the same timepoint was 23.2% (Fig. 1A and B). The cumulative incidence of treated brain metastases in patients with stage III HER2+/HR+, HER2+/HR-, and TNBC at 5 years was 7.5%, 11.2%, and 13.1%, respectively. This was significantly higher than 3.3% in those with stage III HR+/HER2- breast cancer at the same timepoint. The lowest cumulative incidence of treated brain metastasis was 1.2% at 12 years among patients initially diagnosed with stage I/II HR+/HER2- disease (Fig. 2).

**Table 2:** Treated brain metastasis (BrM) incidence proportion by breast cancer subtype and stage.

Stage	Subtype	Total	BrM^a^	No BrM^b^	P Value
All Stages	All Subtypes - n (%)	92,973 (100.0%)	2037 (2.2%)	90,936 (97.8%)	<.0001
	TNBC - n (%)	6751 (7.3%)	386 (5.7%)	6365 (94.3%)	
	HER2+/HR+, - n (%)	7377 (7.9%)	228 (3.1%)	7149 (96.9%)	
	HER2+/HR-, - n (%)	3282 (3.5%)	174 (5.3%)	3108 (94.7%)	
	HR+/HER2-, - n (%)	50,286 (54.1%)	550 (1.1%)	49,736 (98.9%)	
	Unknown - n (%)	25,277 (27.2%)	699 (2.8%)	24,578 (97.2%)	
Stage I	All Subtypes - n (%)	49,718 (100.0%)	307 (.6%)	49,411 (99.4%)	<.0001
	TNBC - n (%)	2169 (4.4%)	49 (2.3%)	2120 (97.7%)	
	HER2+/HR+, - n (%)	3607 (7.3%)	39 (1.1%)	3568 (98.9%)	
	HER2+/HR-, - n (%)	933 (1.9%)	10 (1.1%)	923 (98.9%)	
	HR+/HER2-, - n (%)	31,478 (63.3%)	111 (.4%)	31,367 (99.6%)	
	Unknown - n (%)	11,531 (23.2%)	98 (.8%)	11,433 (99.2%)	
Stage II	All Subtypes - n (%)	31,343 (100.0%)	835 (2.7%)	30,508 (97.3%)	<.0001
	TNBC - n (%)	3084 (9.8%)	151 (4.9%)	2933 (95.1%)	
	HER2+/HR+, - n (%)	2625 (8.4%)	81 (3.1%)	2544 (96.9%)	
	HER2+/HR-, - n (%)	1568 (5.0%)	73 (4.7%)	1495 (95.3%)	
	HR+/HER2-, - n (%)	14,166 (45.2%)	230 (1.6%)	13,936 (98.4%)	
	Unknown - n (%)	9900 (31.6%)	300 (3.0%)	9600 (97.0%)	
Stage III	All Subtypes - n (%)	11,912 (100.0%)	895 (7.5%)	11,017 (92.5%)	<.0001
	TNBC - n (%)	1498 (12.6%)	186 (12.4%)	1312 (87.6%)	
	HER2+/HR+, - n (%)	1145 (9.6%)	108 (9.4%)	1037 (90.6%)	
	HER2+/HR-, - n (%)	781 (6.6%)	91 (11.7%)	690 (88.3%)	
	HR+/HER2-, - n (%)	4642 (39.0%)	209 (4.5%)	4433 (95.5%)	
	Unknown - n (%)	3846 (32.3%)	301 (7.8%)	3545 (92.2%)	

Note: % is row percentage for BrM and no BrM; p value reflects the comparison among the 5 BC subtype groups or 3 stage groups.

Legend: BC = breast cancer; HR = hormone receptor; TNBC = triple negative breast cancer.

a BrM treated with brain surgery and/or radiation (whole brain radiotherapy and/or stereotactic radiosurgery).

b No BrM (no local brain-directed therapy).

### 3.2 Time to brain metastasis

The median TTBM for the entire cohort was 36.9 months (20.4–63.6). The shortest TTBM was seen in patients with stage III HER2+/HR- or TNBC, at 23.3 (14.7 – 40.4) and 18.0 months (12.1–30.8), respectively (Table 3). The Fine-Gray subdistribution hazard model analysis of TTBM with competing risk of death showed higher ratios among patients with stage III TNBC (HR 15.78), HER2+/HR- (HR 13.28) and HER2+/HR+ (HR 9.33) breast cancer compared to patients with stage I/II HR+/HER-disease (Supplemental Table 1).

**Table 3:** Median time from breast cancer diagnosis to brain metastasis (BrM) by breast cancer subtype and stage.

Stage		Breast Cancer Subtype						
	Label	Total	TNBC	HER2+/HR+	HER2+/HR-	HR+/HER2-	Unknown	P Value
Overall cohort	Patient No.	N = 2037	N = 386	N = 228	N = 174	N = 550	N = 699	
	Median (IQR), months	36.9 (20.4-63.6)	24.3 (14.2-39.8)	37.8 (24.3-56.2)	26.1 (16.5-42.0)	45.2 (26.5-69.1)	44.2 (22.8-77.4)	<.0001
Stage I	Patient No.	N = 307	N = 49	N = 39	N = 10	N = 111	N = 98	
	Median (IQR), months	50.8 (30.0-78.4)	37.6 (20.1-54.7)	40.4 (25.3-65.1)	64.2 (51.1-90.2)	43.7 (23.5-76.3)	66.1 (46.5-105.0)	<.0001
Stage II	Patient No.	N = 835	N = 151	N = 81	N = 73	N = 230	N = 300	
	Median (IQR), months	39.3 (23.5-65.6)	29.9 (19.8-51.0)	39.3 (22.7-51.5)	27.9 (18.8-40.9)	48.8 (27.5-68.1)	45.4 (25.3-80.0)	<.0001
Stage III	Patient No.	N = 895	N = 186	N = 108	N = 91	N = 209	N = 301	
	Median (IQR), months	30.8 (17.3-54.7)	18.0 (12.1-30.8)	35.9 (23.6-56.2)	23.3 (14.7-40.4)	44.1 (25.3-68.4)	34.8 (18.9-66.5)	<.0001
Stages I and II	Patient No.	N = 1142	N = 200	N = 120	N = 83	N = 341	N = 398	
	Median (IQR), months	41.9 (24.5-67.4)	30.4 (20.0-51.8)	39.6 (24.8-56.1)	29.9 (18.8-51.1)	47.7 (27.3-69.5)	53.1 (28.9-90.5)	<.0001

Legend: BC = breast cancer; HR = hormone receptor; IQR = interquartile range; No. = number; TNBC = triple negative breast cancer.

### 3.3 Survival after treatment for brain metastasis

The median survival time from treated brain metastases to death due to any cause in the full cohort was 6.8 (2.2-20.0) months. Patients with HER2+ breast cancer had a longer survival [15.2 (5.3-44.5) months for HER2+/HR+ and 15.3 (4.0-31.7) months for HER2+/HR-] compared to those with TNBC [3.9 (1.9-9.7) months] or HR+/HER2- breast cancer [7.3 (2.0-23.6) months] (Table 4).

**Table 4:** Median survival time from treatment of brain metastasis (BrM) to death by breast cancer subtype and stage.

Stage		BC Subtype						
		Total	TNBC	HER2+/HR+	HER2+/HR-	HR+/HER2-	Unknown	P Value
Stages I, II, III	Patient No.	N = 2037	N = 386	N = 228	N = 174	N = 550	N = 699	
	Median (IQR), months	6.8 (2.2-20.0)	3.9 (1.9-9.7)	15.2 (5.3-44.5)	15.3 (4.0-31.7)	7.3 (2.0-23.6)	5.5 (2.0-16.1)	<.0001
Stage I	Patient No.	N = 307	N = 49	N = 39	N = 10	N = 111	N = 98	
	Median (IQR), months	8.6 (2.7-56.8)	5.4 (2.6-11.7)	10.7 (4.2-42.1)	6.4 (1.2-21.3)	15.6 (2.3-83.2)	7.8 (2.8-35.1)	.0630
Stage II	Patient No.	N = 835	N = 151	N = 81	N = 73	N = 230	N = 300	
	Median (IQR), months	6.8 (2.2-18.5)	4.9 (2.1-11.6)	12.7 (4.8-36.1)	17.6 (4.8-32.8)	6.7 (2.0-20.6)	5.3 (2.0-13.0)	<.0001
Stage III	Patient No.	N = 895	N = 186	N = 108	N = 91	N = 209	N = 301	
	Median (IQR), months	6.2 (2.1-18.4)	3.3 (1.6-9.0)	16.2 (6.5-53.3)	15.0 (3.9-27.9)	6.2 (2.0-18.1)	5.4 (2.0-16.2)	<.0001
Stages I, II	Patient No.	N = 1142	N = 200	N = 120	N = 83	N = 341	N = 398	
	Median (IQR), months	7.2 (2.3-21.4)	5.3 (2.3-11.6)	12.7 (4.8-42.1)	17.5 (4.2-31.7)	8.0 (2.1-32.7)	5.7 (2.1-16.0)	<.0001

Legend: BC = breast cancer; HR = hormone receptor; IQR = interquartile range; No. = number; TNBC = triple negative breast cancer.

## 4 Discussion

Among 92,973 patients with stage I–III breast cancer, the cumulative incidence of treated brain metastases was 2.8% at 12 years after breast cancer diagnosis. Patients with stage III HER2+/HR+, HER2+/HR−, and TNBC had a 12-year cumulative incidence of treated brain metastases of 11.8%, 14.3%, and 13.4%, respectively. Importantly, the median TTBM for patients with stage III HER2+/HR− or TNBC was less than 2 years from the time of initial diagnosis. These numbers are similar to other primary tumors where brain imaging is considered part of staging investigations, like lung cancer (lifetime incidence of brain metastases 14.1% across all stages [17]) and melanoma (lifetime incidence of brain metastases 16.7% among those with stage III disease [18]).

A population-based study from the Flatiron Health database in the United States of America analyzed 18,075 patients with metastatic breast cancer, including 12,090 (66.9%) with recurrent disease, to describe the incidence of brain metastases by line of therapy received in the metastatic setting [19]. Recognizing that this study was not designed to investigate the prevalence of brain metastases among patients with early-stage breast cancer, the findings show a high prevalence of brain metastases among patients with TNBC and HER2+/HR- breast cancer within one year of initiating therapy for metastatic disease. This aligns with our findings and suggests an early and relatively high incidence of brain metastases in patients with HER2+ and TNBC subtypes. In the Surveillance, Epidemiology, and End Results (SEER) database, the incidence of brain metastases at initial breast cancer diagnosis was reported among 394,153 patients [20]. The overall incidence of brain metastases was .67%, with higher incidences of 1.17% for HER2+ and .71% for TNBC subtypes [20]. However, as noted by the authors, data regarding brain metastases is not reliably recorded in SEER and cumulative incidence of brain metastases over time was not reported [20].

In our cohort, brain metastases occurred more frequently among patients with HER2+ or TNBC. Pivotal trials for early HER2+ breast cancer, such as the HERA [21] and KATHERINE [22] trials, reported lower rates of central nervous system (CNS) as site of first relapse: 2% in HERA (median TTBM of 1.26 years) and 5.9% in the TDM-1 arm of KATHERINE (median TTBM of 17.5 months). For TNBC, the KEYNOTE-522 trial reported the incidence of brain metastases as a first site of metastatic relapse among 1174 patients with stage II–III TNBC at 2.3% and 3.3% in the intervention and control arms, respectively [23]. In all of these trials, the true incidence of brain metastases is likely underestimated given that brain imaging surveillance is not typically performed in the absence of symptoms. Also, comparisons with our study are limited as we cannot state whether the brain was the first site of metastatic relapse. A prospective cohort study (NCT06247449) is ongoing to assess the incidence of asymptomatic brain metastases in patients with stage IIb–III HER2+ or TNBC subtypes.

Our study highlights differences in some baseline characteristics between patients with treated brain metastases and those without. Advanced stage and breast cancer subtype (HER2+ or TNBC), as expected, were the most important risk factors for development of brain metastases. But age (53.0 vs. 61.0, p < 0.0001) and high comorbidity index (as assessed by Elixhauser scores ≥5) (9.2% vs. 13.2%, p < 0.0001) were significantly lower in patients with treated brain metastases. These findings suggest that younger patients with fewer comorbidities may be at higher risk for brain metastases, which could be explained by a higher proportion of HER2+ and TNBC in the younger population [24,25] and the well-established association between these breast cancer subtypes and brain metastases [26].

A deeper understanding of the primary tumor biology among patients who develop brain metastases is of interest. In non-small cell lung cancer, tissue-based next-generation sequencing (NGS) [27] and liquid-based biomarkers (methylated cell-free DNA sequenced in plasma) [28] have shown promise in identifying patients at risk of developing brain metastases. In addition, a large study assessing molecular profiling results of patients with breast cancer (obtained using the FOUNDATION™ test), markers of homologous recombination deficiency and others associated with sensitivity to immune checkpoint inhibition were significantly more common in brain metastases compared to primary tumors and/or extra-cranial sites of metastatic disease [28]. Such genomic analyses combined with epidemiological data could enable the design of clinical trials to evaluate treatment escalation, de-escalation, and prevention strategies tailored to those at greatest risk of developing brain metastases.

Finally, the overall survival of patients after treatment for brain metastases diagnosis was short at 6.8 months, which aligns with data from both the ESME (Epidemiological Strategy and Medical Economics) and BMBC (Brain Metastasis in Breast Cancer) registries at 7.9 [29] and 7.4 [30] months, respectively; as expected, overall survival varied by breast cancer subtype across relevant studies. The shortest survival was observed among patients with TNBC (3.9 months) and the longest among those with HER2+ breast cancer (∼15 months). This is consistent with previous data [[2], [31]], and can be partially explained by the fact that patients with HER2+ brain metastases benefit from a broader range of therapies with intracranial efficacy [32]. However, modern therapies with CNS activity, such as the HER2CLIMB regimen [[32], [33]] and trastuzumab deruxtecan [[32], [34]] were not publicly available in Canada before 2021 and 2022, respectively and, therefore, not routinely prescribed during the timeframe of this study. Hence, the survival of patients with HER2+ brain metastases is expected to be longer in a more contemporary cohort.

An important limitation of this study is our inability to determine extracranial disease status at the time of treatment for brain metastases, preventing us from assessing whether the brain was the first or a subsequent site of metastatic recurrence. Although infrequent, .1% of patients with stage III triple negative or HER2+ breast cancer (0% among those with stage I/II disease) were found to have treated brain metastasis within 3 months of their breast cancer diagnosis, raising the possibility that they had undiagnosed metastatic disease at the time of initial presentation, or were incorrectly coded as having early-stage disease. Additionally, as in prior studies using ICES data, we lacked detailed information on intracranial disease burden, type of CNS involvement (parenchymal vs. leptomeningeal) and cause of death. The absence of granular data also limited our assessment of response to neoadjuvant therapy, systemic treatment modalities, neurologic symptoms, and cognitive outcomes. Furthermore, a brain metastases diagnosis was inferred from brain surgery for malignancies and/or radiotherapy, as no specific diagnostic code for brain metastases exists in the ICES database. Hence, patients receiving palliative care only or systemic therapy only would not have been captured as having brain metastases. However, during the time period of this study, intra-cranial efficacy of HER2-directed systemic therapy had not yet been established so treatment of brain metastases with systemic therapy alone was not considered standard practice. Moreover, a prior Ontario study found that approximately 91% of breast cancer patients with brain metastases received radiotherapy, suggesting that the great majority of brain metastases would be captured using our approach [7]. Finally, of the 476 patients who underwent brain surgery, only very few (n = 7) had neurosurgical procedures that could potentially have been performed for non-oncologic indications. As patients with a secondary malignancy were excluded, individuals diagnosed with a primary CNS malignancy or other metastatic solid tumor after brain resection would have been excluded.

## 5 Conclusion

Up to 13% of patients with stage III HER2+ or TNBC received treatment for brain metastases within 5 years of diagnosis of early-stage breast cancer. The true incidence of asymptomatic brain metastases in this population is likely higher as routine brain metastases screening is not performed for patients with early-stage breast cancer. These findings support prospective studies of risk-stratified screening for asymptomatic brain metastases in patients with early-stage breast cancer.

## CRediT authorship contribution statement

**Katarzyna J. Jerzak:** Writing – review & editing, Writing – original draft, Supervision, Resources, Project administration, Methodology, Investigation, Formal analysis, Data curation, Conceptualization. **Italo Fernandes:** Writing – review & editing, Writing – original draft, Investigation. **Hany Soliman:** Writing – review & editing, Methodology, Investigation, Conceptualization. **Bo Zhang:** Writing – review & editing, Methodology, Formal analysis, Data curation. **Refik Saskin:** Writing – review & editing, Methodology, Investigation, Formal analysis. **Geoffrey Liu:** Writing – review & editing, Methodology, Investigation. **Sunit Das:** Writing – review & editing, Methodology, Investigation. **Arjun Sahgal:** Writing – review & editing, Methodology, Investigation. **Kelvin KW. Chan:** Writing – review & editing, Methodology, Investigation. **Monika Kryzanowska:** Writing – review & editing, Methodology, Investigation. **Rania Chehade:** Writing – review & editing, Writing – original draft, Methodology, Investigation, Data curation.

## Data availability statement

The data underlying this article cannot be shared publicly due to ICES policy. De-identified aggregate data will be shared on reasonable request to the corresponding author, pending ICES permission.

## Funding

This study was not funded.

## Declaration of competing interest

**K.J.J** has been a consultant, speaker, or advisory board member for Amgen, AstraZeneca, Apo Biologix, BioCon, Daiichi Sankyo, Eli Lilly, Esai, Genomic Health, Gilead Sciences, Knight Therapeutics, Merck, Myriad Genetics, Novartis, Organon, Pfizer, Roche, and Viatris; has received research funding from AstraZeneca, Daiichi Sankyo, Eli Lilly, and Pfizer; and has received drug supply from Pfizer and BioCon for an investigator initiated clinical trial. **R.C.** has received honoraria from AstraZeneca, Eli Lilly, GSK and Merck. **A.S.** reports consulting services' fees, honorarium, or both for past educational seminars for Varian Medical Systems, Elekta, AstraZeneca, Medtronic Kyphon, and BrainLAB; and research grants from Elekta and Varian Medical Systems. Remaining authors do not have any relevant conflicts of interest to disclose.
