# Supplementary material for: Incidence of treated brain metastases among patients with stage I-III breast cancer: A population-based study
Source: Breast. 2026 Feb 13;86:104723. doi: 10.1016/j.breast.2026.104723 (PMC12937143; doi:10.1016/j.breast.2026.104723)
Supplement: Multimedia component 1 [file mmc1.docx]

**Supplement Figure 1.** Flow diagram of study population: stage I–III breast cancer, Ontario, 2009–2021.

Patients identified at the Institute of Clinical Evaluative Sciences with a new stage I – III breast cancer between January 1^st^, 2009 and December 31^st^, 2021

**N= 137,217**

Patients included in the analysis: after exclusion criteria applied:

**N=92,973**

**Patients excluded**

18,388 had other cancer diagnosis before index date

8,456 patients had a subsequent non-breast cancer on or after the index date

747 were under the age of 18 years

721 were ineligible for OHIP

628 died on or before the index date

45 were non-Ontario residents

| **Supplemental Table 1. Fine-Gray Subdistribution Hazard Model Analysis of Time from Breast Cancer Diagnosis to Treated Brain Metastasis with Competing Risk of Death among All BC Patients by BC Subtype and Stage (Brain metastases events n=2,037, Competing Deaths n=14,937, Patients n=92,973)** | | | | | |
| --- | --- | --- | --- | --- | --- |
|  |  | **Crude Analysis** | | **Adjusted Analysis** | |
| **Variable** | **Label** | **Hazard Ratio (95% CI)** | **P value** | **Hazard Ratio (95% CI)** | **P value** |
| BC Subtype and Stage | TNBC Stages I/II vs. HR+/HER2- Stages I/II | 4.97 (4.18-5.92) | <.0001 | 4.31 (3.61-5.14) | <.0001 |
|  | TNBC Stage III vs. HR+/HER2- Stages I/II | 18.23 (15.20-21.86) | <.0001 | 15.78 (13.11-18.99) | <.0001 |
|  | HER2+/HR+ Stages I/II vs.HR+/HER2- Stages I/II | 2.61 (2.12-3.21) | <.0001 | 2.21 (1.79-2.72) | <.0001 |
|  | HER2+/HR+ Stage III vs. HR+/HER2- Stages I/II | 11.90 (9.59-14.78) | <.0001 | 9.33 (7.50-11.59) | <.0001 |
|  | HER2+/HR- Stages I/II vs. HR+/HER2- Stages I/II | 4.50 (3.54-5.72) | <.0001 | 3.79 (2.98-4.83) | <.0001 |
|  | HER2+/HR- Stage III vs. HR+/HER2- Stages I/II | 16.03 (12.68-20.27) | <.0001 | 13.28 (10.47-16.83) | <.0001 |
|  | HR+/HER2- Stage III vs. HR+/HER2- Stages I/II | 5.51 (4.64-6.55) | <.0001 | 4.79 (4.03-5.69) | <.0001 |
|  | Unknown Subtype Stages I/II/III vs. HR+/HER2- Stages I/II | 2.92 (2.56-3.33) | <.0001 | 2.16 (1.73-2.70) | <.0001 |
| Age at BC Diagnosis | Continuous (per 10 year increment) | 0.65 (0.62-0.67) | <.0001 | 0.70 (0.68-0.72) | <.0001 |
| Income Quintile and Rurality | First Quintile vs. 5th (Highest) Quintile | 0.87 (0.75-1.01) | 0.0757 | 0.87 (0.74-1.01) | 0.0642 |
|  | 2nd Quintile vs. 5th (Highest) Quintile | 0.90 (0.78-1.04) | 0.1642 | 0.90 (0.78-1.04) | 0.1504 |
|  | 3rd Quintile vs. 5th (Highest) Quintile | 1.08 (0.94-1.24) | 0.2810 | 1.02 (0.89-1.17) | 0.8099 |
|  | 4th Quintile vs. 5th (Highest) Quintile | 0.99 (0.86-1.14) | 0.8670 | 0.93 (0.81-1.07) | 0.3341 |
|  | Rural Residence vs. 5th (Highest) Quintile | 0.92 (0.78-1.08) | 0.3193 | 1.00 (0.85-1.17) | 0.9594 |
|  | Misssing vs. 5th (Highest) Quintle | 1.74 (0.77-3.93) | 0.1805 | 1.75 (0.77-4.00) | 0.1827 |
| Elixhauser Index | Score < 0 vs. 0 | 1.07 (0.96-1.21) | 0.2324 | 0.99 (0.88-1.11) | 0.8621 |
|  | Score 1-4 vs. 0 | 1.08 (0.97-1.20) | 0.1777 | 1.08 (0.97-1.20) | 0.1467 |
|  | Score >=5 vs. 0 | 0.70 (0.60-0.82) | <.0001 | 0.95 (0.81-1.11) | 0.5238 |
| Index Year at BC Diagnosis | 2010 vs. 2009 | 0.94 (0.77-1.14) | 0.5036 | 0.94 (0.78-1.14) | 0.5327 |
|  | 2011 vs. 2009 | 0.86 (0.70-1.04) | 0.1243 | 0.87 (0.71-1.06) | 0.1578 |
|  | 2012 vs. 2009 | 0.90 (0.74-1.10) | 0.3002 | 0.77 (0.58-1.01) | 0.0631 |
|  | 2013 vs. 2009 | 1.03 (0.85-1.24) | 0.7933 | 0.92 (0.70-1.20) | 0.5228 |
|  | 2014 vs. 2009 | 0.84 (0.69-1.03) | 0.0899 | 0.74 (0.56-0.97) | 0.0292 |
|  | 2015 vs. 2009 | 0.86 (0.71-1.05) | 0.1432 | 0.77 (0.58-1.01) | 0.0619 |
|  | 2016 vs. 2009 | 0.79 (0.64-0.97) | 0.0255 | 0.72 (0.54-0.95) | 0.0205 |
|  | 2017 vs. 2009 | 0.74 (0.59-0.91) | 0.0051 | 0.68 (0.51-0.90) | 0.0071 |
|  | 2018 vs. 2009 | 0.61 (0.48-0.77) | <.0001 | 0.62 (0.46-0.85) | 0.0026 |
|  | 2019 vs. 2009 | 0.52 (0.40-0.67) | <.0001 | 0.54 (0.39-0.75) | 0.0002 |
|  | 2020 vs. 2009 | 0.71 (0.55-0.93) | 0.0109 | 0.71 (0.51-0.98) | 0.0386 |
|  | 2021 vs. 2009 | 0.54 (0.39-0.75) | 0.0002 | 0.56 (0.39-0.80) | 0.0016 |

Legend: BC= breast cancer; HR = hormone receptor; IQR= Linterquartile range; TNBC= triple negative breast cancer.
